# Determinants of antenatal care utilization – contacts and screenings – in Sao Tome & Principe: a hospital-based cross-sectional study

**DOI:** 10.1186/s13690-023-01123-1

**Published:** 2023-06-16

**Authors:** Alexandra Vasconcelos, Swasilanne Sousa, Nelson Bandeira, Marta Alves, Ana Luísa Papoila, Filomena Pereira, Maria Céu Machado

**Affiliations:** 1 Unidade de Clínica Tropical - Global Health and Tropical Medicine (GHTM), Instituto de Higiene e Medicina Tropical (IHMT), Universidade Nova de Lisboa, Lisbon, Portugal; 2 Department of Pediatrics, Hospital Dr. Ayres de Menezes, São Tomé, Sao Tome and Principe; 3 Department of Obstetrics and Gynecology, Hospital Dr. Ayres de Menezes, São Tomé, Sao Tome and Principe; 4 CEAUL, NOVA Medical School/Faculdade de Ciências Médicas, Universidade Nova de Lisboa, Lisbon, Portugal; 5 Faculdade de Medicina de Lisboa, Universidade de Lisboa, Lisboa, Portugal

**Keywords:** Antenatal care utilization, Sao Tome & Principe, Pregnant women, Newborn mortality, Antenatal screenings, Adequate antenatal care utilization

## Abstract

**Background:**

Sao Tome & Principe (STP) has a high peri-neonatal mortality rate and access to high-quality care before childbirth has been described as one of the most effective means of reducing it. The country has a gap in the coverage-content of antenatal care (ANC) services that must be addressed to better allocate resources to ultimately improve maternal and neonatal health. Therefore, this study aimed to identify the determinants for adequate ANC utilization considering the number and timing of ANC contacts and screening completion.

**Methods:**

A hospital based cross-sectional study was undertaken among women admitted for delivery at Hospital Dr. Ayres de Menezes (HAM). Data were abstracted from ANC pregnancy cards and from a structured face-to-face interviewer-administered questionnaire. ANC utilization was classified as partial vs adequate. Adequate ANC utilization was defined as having ANC 4 or more contacts, first trimester enrolment plus one or more hemoglobin tests, urine, and ultrasound. The collected data were entered into QuickTapSurvey and exported to SPSS version 25 for analysis. Multivariable logistic regression was used to identify determinants of adequate ANC utilization at *P*-value < 0.05.

**Results:**

A total of 445 mothers were included with a mean age of 26.6 ± 7.1, an adequate ANC utilization was identified in 213 (47.9%; 95% CI: 43.3–52.5) and a partial ANC utilization in 232 (52.1%; 95% CI: 47.5–56.7). Age 20–34 [AOR 2.27 (95% CI: 1.28–4.04), *p* = 0.005] and age above 35 [AOR 2.5 (95% CI: 1.21–5.20), *p* = 0.013] when comparing with women aged 14–19 years, urban residence [AOR 1.98 (95% CI: 1.28–3.06), *p* < 0.002], and planned pregnancy [AOR 2.67 (95% CI: 1.6–4.2), *p* < 0.001] were the determinants of adequate ANC utilization.

**Conclusion:**

Less than half of the pregnant women had adequate ANC utilization. Maternal age, residence and type of pregnancy planning were the determinants for adequate ANC utilization. Stakeholders should focus on raising awareness of the importance of ANC screening and engaging more vulnerable women in earlier utilization of family planning services and choosing a pregnancy plan, as a key strategy to improve neonatal health outcomes in STP.

**Table:** 

**Text box 1. Contributions to the literature**
• Achieving a 90% coverage rate of 4+ ANC contacts by 2025 is one of the targets of the 90/90/80/80 milestones. Low-resource settings, such as Sao Tome & Principe, are far behind with major gaps in the quality of ANC coverage-content.
• Missing out ANC screenings represents losing the prevention and treatment of adverse outcomes, such as low-birthweight, prematurity, sepsis, and stillbirths.
• This study adds to the literature as it addresses the determinants of ANC utilization, classified as adequate or partial based on the number of ANC contacts, timing of first booking plus the completion of hemoglobin, urine tests and obstetric ultrasound.

## Background

Poor antenatal healthcare (ANC) utilization is an international public health challenge especially in low-resource countries, with prejudice toward the health of the expectant mother and the unborn child [1, 2]. Access to a quality ANC service remains a major challenge in efforts to reduce neonatal morbidity and mortality [1].

Antenatal care status is typically characterized only by the number or timing of visits or ANC contacts [3]. However, these optimum numbers of attendances have been changing in recent decades. Initially, the World Health Organization (WHO) suggested numerous ANC visits (7–16), a burden in resource-constrained settings; then, in 2001, Focused Antenatal Care (FANC) reduced it to a minimum of four visits [4, 5]. Later, studies concluded that eight ANC contacts could reduce perinatal deaths by up to eight per 1000 births when compared to four visits [6]. Consequently, in 2016, the WHO established eight ANC contacts to reduce perinatal mortality and improve mothers’ experience of care [7].

Evidence-based screenings are also endorsed by the WHO, including a core set of interventions such as blood pressure measurement, iron tablet supplementation, tetanus toxoid vaccination, urine testing, hemoglobin test, and rapid tests for sexually transmitted diseases [1]. These screenings are able to identify unrecognized diseases or conditions in apparently healthy pregnant women by means of tests that can be applied rapidly and easily to the target population with irrefutable benefits in maternal and neonatal health [1, 8].

Nonetheless, there is a gap in the actual use and implementation of these ANC screening practices, mainly in sub-Saharan African (SSA) countries [8].

The magnitude of the gap varies, with greater concerns reported in relation to underuse compared to overuse, mainly in screening completion [8].

Factors linked to maternal ANC utilization, are usually grouped into community-level features, sociodemographic and pregnancy-related characteristics, media exposure and availability of maternal health services [2]. Effectively, the determinants of ANC utilization are maternal education, women’s employment, marital status, husband’s education, household income, availability, cost, and pregnant women’s history of obstetric complications [8, 1]. These factors can influence the ANC use of these women either positively or negatively [1].

Sao Tome & Principe (STP), a sub-Sharan Island country in the Gulf of Guinea, is characterized by a total fertility rate of 4.4 (number of births per woman) and a considerable unmet need for contraception of 33.7% [9]. Although ANC contacts are available free of charge to all women, most screenings have high costs to pregnant women, limiting their completion and the quality of care provided [9].

The country already reached two out of four 90/90/80/80 targets and milestones for maternal and newborn health, with 95.4% women receiving the service of a skilled birth attendant at birth and 91% newborns obtaining first postnatal care within the first 24 h after birth [9, 10]. Missing the target of 90% coverage of ANC 4 or more contacts and the 80% availability at the districts of critical emergency care for women with complications and for small and sick newborns [9, 10]. Despite all these achievements, at the start of this research, neonatal mortality in STP was high, with 22 newborn babies dying per 1000 live births and 22 stillbirths per 1000 live births [11].

Most studies about ANC in Sub-Saharan countries dichotomize the outcome variable ANC into utilized and not utilized—only based on the total number of visits/contacts—disregarding whether the WHO´s recommended screenings were completed [12, 13]. Missing out on these evidence-based screening interventions represents losing the prevention or treatment of maternal anemia, bacteriuria, puerperal sepsis, reduction of the risk of low-birth-weight neonates, preterm birth, neonatal sepsis, and stillbirths [7, 12, 13]. ANC optimization has a key role in reducing of neonatal mortality and adverse birth outcomes and improving overall neonatal health outcomes [2]. Therefore, in the present study, we decided to go further on and assess essential ANC screening per pregnant woman (obstetric ultrasound, hemoglobin, and urine tests), in addition to the total number of ANC contacts [14]. We now aim to understand pregnant women´s profile depending on the type of ANC utilization with the purpose of contributing to the development of a strategy to improve maternal and newborn health as endorsed in the post-2015 Sustainable Development Goals of no more than 12 neonatal deaths per 1000 livebirths for Sao Tome and Príncipe [15].

## Material and methods

### Study design and period

An institutional-based cross-sectional analytical study was conducted from July 2016 to November 2018 in the only country´s hospital, Hospital Dr. Ayres de Menezes (HAM).

### Study context

The archipelago of STP has two islands, with a total land surface of approximately 1,001 km^2^, 859 km^2^ of which for Sao Tome and 142 km^2^ for Principe with approximately 200.000 inhabitants [9, 10]. All six districts have maternity units: Lembá, Lobata, Caué, Me-Zochi, Principe, Água Grande (HAM), exception for the district of Cantagalo [9, 10]. Complete obstetric services (cesarean section and blood transfusion) are only available at HAM. There is no health insurance policy in the country or any private maternity units. ANC contacts are provided by nurses [7, 9, 16]. The ANC services offered are documented on women´s antenatal pregnancy card, which remains with the pregnant women for taking it to the maternity unit at the time of the babies` delivery.

### Study population

This study was conducted in HAM which has a maternity unit responsible for 82,4% of all deliveries in the country [9, 10]. An estimated total number of 4500 births was registered during the study period.

All pregnant women admitted to the HAM maternity unit for delivery constituted the source population, whereas the study populations were selected pregnant women admitted to the HAM maternity unit during the study period.

The eligibility criteria for participants were as follows: 1) all women admitted to the hospital for delivery with a gestational age of 28 weeks or more, 2) prepartum adolescents and illiterate women who had obtained permission from their parent(s) or legal guardian(s) to participate in the study and 3) those who gave birth outside the hospital but were later admitted at HAM on the day of birth.

The exclusion criteria included the following: 1) women with induced termination of pregnancy for medical reasons, 2) adolescent or illiterate mothers who had not obtained permission from their parents or legal guardians to participate in the study, 3) women without an ANC pregnancy card and 4) those missing information on the timing of first ANC contact.

Of the 534 eligible participants, 89 women were excluded (16 for being discharged before conducting the face-to-face interview, 7 due to not having an ANC pregnancy card and 66 due to missing information on the timing of first ANC contact). A total of 445 pregnant women were included in this study.

### Sample size determination and sampling procedure

The sample size was calculated using Raosoft software (http://www.raosoft.com/samplesize.html). Using a minimum sample of 10% of the population, validated by the software, placed the sample right dimension between 355 (95%) and 579 (99%) confidence. This size was also supported by PASS software (https://www.ncss.com/software/pass/). It was possible to collect 445 women, which gave some comfort at this level. Eligible cases were selected randomly until the required sample size was achieved.

### Data collection tools/methods

Data on this study were gathered through a structured face-to-face interviewer-administered questionnaire plus data abstraction from the pregnant women´s ANC pregnancy card. A structured questionnaire adapted from other similar studies was used [1, 8, 17, 18]. Issues covered in each questionnaire included data on age, residence, maternal education, partner education, maternal occupational status, marriage, planned pregnancy, and previous contraceptive utilization. Some questions had dichotomous answers, for example, type of pregnancy planning (planned or unplanned) and contraceptive use (previous use or never use). In addition to the interview, the researcher abstracted clinical data by reviewing the mother´s ANC pregnancy card to collect information regarding screening tests performed, parity, number of ANC contacts, and timing of first booking.

### Data quality control

The questionnaires were administered in Portuguese, the national language in Sao Tome & Principe. Five percent of the questionnaire was pretested in the same study area one month before data collection, and modification was made based on the pretest result. Administered questionnaires were checked for completeness and consistency. Supervision of data collection and continuous follow-up were provided by the supervisors. The main investigator (a pediatrician, PhD researcher) executed and was responsible for all activities as follows: obtaining consent and enrollment of the mothers, data collection from ANC pregnancy cards, face-to-face interviews, and performing all data collection entry into the survey app.

### Variables

#### Outcome variable

The outcome variable of interest was the utilization of ANC services. This utilization was classified as adequate or partial, based on the number of ANC contacts and first booking in the first trimester as well as the completion of hemoglobin, urine tests and obstetric ultrasound.

**Adequate ANC utilization** was defined when **all** the following interventions were achieved: 1) attended ANC four or more times, 2) first booking during the first trimester, 3) had at least done one ultrasound, 4) had at least one urine test and 5) performed no less than one hemoglobin test.

**Partial ANC utilization** category involved the remaining pregnant women, namely, those who attended antenatal clinic one or more times regardless of, whether they had or not, an obstetric ultrasound, an urine or any hemoglobin tests.

#### Explanatory variables

In accordance with previous knowledge, the variables included in this study that could potentially be associated with ANC service utilization were age, residence, maternal education, husband/partner education, maternal occupational status, marriage, planned pregnancy, contraceptive utilization, and parity [13, 14, 19].

Age was categorized as 14–19 years, 20–34 years and 35 years and above. Women’s education level has been included as a categorical variable ranging from no education, primary education, secondary and higher education, the same criteria for the baby’s father’s education. Household characteristics were characterized as having sanitation (toilet facilities or latrine) or not. Residence was grouped into urban and rural. Urban residence for women living at the capital city (Água Grande) and rural residence in all other districts. Employment status (working or not working) was defined as working for those who engaged in one economic activity. Parity was categorized as nulliparous, one to four parity and grand multipara (five or more). Marital status considered the two categories married/union vs single/never married. Women who were formally married or those who reported being in a union (cohabiting with a partner) were categorized as married/union.

### Data management and statistical analysis

Data entry for this study into the app survey was first cleaned by creating a data entry field in Excel, and all categorical responses were checked for completeness and accuracy and to eliminate errors and inconsistent data. Data were further analyzed using the Statistical Package for the Social Sciences for Windows, version 25.0 (IBM Corp. Released 2017. IBM SPSS Statistics for Windows, Version 25.0. Armonk, NY: IBM Corp.).

Data analysis in this study was carried out in two stages. The first stage involved pooling data for descriptive statistics with categorical variables presented as frequencies and percentages, and quantitative variables presented as the mean and standard deviation. The second stage involved a univariable analysis to identify the candidate variables for the multivariable analysis (with a *p* value < 0.25). In all these analyses, logistic regression models for binary response were applied. Linearity in the logit assumption of age was verified with generalized additive regression models. The crude odds ratios (COR) and adjusted odds ratios (AOR) with corresponding 95% confidence intervals (95% CI) were then estimated. The level of significance α = 0.05 was considered. The goodness-of-fit of the multivariable model was checked with the Hosmer–Lemeshow test.

### Ethics approval and consent to participate

The study was approved and consented to by the Ministry of Health of Sao Tome and Principe and by the main board of Hospital Dr. Ayres de Menezes, since at the time the study protocol was submitted there were no ethics committees in Sao Tome and Principe. Only recently has the country National Ethics Committee been appointed. Previously, study analysis and approval were done by the Ministry of Health and the institution where the study was to be performed, which is what we have done. All methods were performed in accordance with the relevant guidelines and regulations in practice. Written informed consent was obtained from all participants (or their parent or legal guardian in the case of adolescents under 16) after the purpose of the research was explained orally by the main investigator. Additionally, if the mother was illiterate and unable to write, oral consent was given in the presence of at least one witness, usually the ward nurse´s complementarity to the mother´s fingerprint mark in the consent form. Participation in the survey was voluntary, as participants could decline to participate at any time during the study. The anonymity and safety of participants were ensured.

## Results

### Sociodemographic characteristics of the study participants

Table 1 shows the sociodemographic characteristics of the participants. Of the 445 pregnant women included, 278 (62.5%) were aged 20 to 34, and the mean age was 26.6 ± 7.1 years (minimum age 14 and maximum 43 years). Three hundred seventy (84.9%) participants were married, 250 (56.6%) ended school with a primary level of education, 239 (54.2%) lived in a house with sanitation, and 307 (69.5%) were unemployed.

**Table 1:** Sociodemographic, community-level and pregnancy-related characteristics for pregnant women (445) admitted to Hospital Dr Ayres de Menezes, Sao Tome & Principe (2016–2018)

Variables	Category	Frequency	Percentage
Age	14–19	92	20.7
	20–34	278	62.5
	35 +	75	16.9
Education	none	14	3.1
	primary	250	56.2
	secondary	148	33.3
	higher	33	7.4
Employment	not working	307	69.5
	working	135	30.5
Marital status	union/married	370	84.9
	single	66	15.1
Father education	none	10	3.3
	primary	146	47.9
	secondary	114	37.4
	higher	35	11.5
Residence	urban	202	46.2
	rural	235	53.8
Household sanitation	open defecation	202	45.8
	sanitation	239	54.2
Parity	0	136	30.6
	1–4	271	60.9
	5 +	38	8.5
Pregnancy planning	unplanned	252	68.9
	planned	114	31.1
Contraceptive previous use	yes	90	24.5
	no	278	75.5

### Pregnancy-related characteristics

A parity of one to four was observed in 271 (60.9%) pregnant women, with 136 (30.6%) of the participants being nulliparous. Unplanned pregnancy was reported in 252 (68.9%) cases, and previous contraceptive use occurred in 90 (24.5%) of the participants.

### Community-level features

Residency in an urban area was assessed for 202 (46.2%) participants, and 235 (53.8%) were living in rural districts, namely, 129 (30%) in Mé-Zochi, 48 (10.8%) in Cantagalo, 25 (5.6%) in Lobata, 17 (3.8%) in Lembá, 9 (2%) in Caué and 7 (1.6%) in Principe Island.

### Antenatal care utilization: adequate versus partial

Of the 445 participants, 378 (84.9%) had four or more ANC visits, 268 (60%) had a first booking during the first trimester, 214 (48%) pregnant women had at least one obstetric ultrasound, 280 (62.9%) had one urine test and 294 (66%) had a hemoglobin test.

The results in Table 2 show the criteria for ANC service utilization classification (adequate versus partial).

**Table 2:** ANC service utilization among pregnant women enrolled (445)

Variables	Category	Frequency	Percentage
ANC 4 or more contacts	yes	378^a^	84.90%
	no	67	15.10%
Early booking in the 1^st^ trimester	yes	268^a^	60%
	no	177	40%
Obstetric ultrasound (at least one)	yes	214^a^	48%
	no	231	52%
Urine test (at least one)	yes	280^a^	62.90%
	no	165	37.10%
Hemoglobin test (at least one)	yes	294^a^	66%
	no	151	34%

^a^Adequate ANC utilization was identified in 213 (47.9%) pregnant women defined for those who had **all** the following criteria: ANC 4 or more contacts ( +) first booking during the first trimester ( +) at least one ultrasound ( +) at least one urine test ( +) and no less than one hemoglobin test

Adequate ANC service utilization was identified for 213 (47.9%; 95% CI: 43.3–52.5) pregnant women. Partial ANC service utilization was defined for the remaining 232 (52.1%; 95% CI: 47.5–56.7) women.

Of note, from the adequate ANC utilization group, a “complete” ANC utilization was found in 22 pregnant women who had all the following: attended ANC clinic eight or more times, first booking in the first trimester, had at least one ultrasound, had at least two urine tests, and performed no less than two hemoglobin tests.

### Antenatal care utilization – Univariable analysis

The results of the univariable analysis (Table 3) showed a statistically significant association between adequate ANC service utilization and the age of the mother, residence, household sanitation and pregnancy planning.

**Table 3:** Determinants associated with ANC utilization in univariable logistic regression analyses among pregnant women admitted at Hospital Dr Ayres de Menezes, Sao Tome & Principe

	ANC service utilization			
	Adequate	Partial	COR [95% CI]	*p* value
	*n* = 213 (47.9%)	*n* = 232 (47.9%)		
Age				
14–19	30 (32.6%)	62 (67.4%)	1.00	
20-34	145 (52.2%)	133 (47.8%)	2.25 [1.37 to 3.70]	**0.001**
35+	33 (50.7%)	37 (49.3%)	2.12 [1.13 to 3.98]	**0.019**
Education				
none	7 (50%)	7 (50%)	1.00	
Primary	104 (41.6%)	146 (58.4%)	0.71 [0.243 to 2.09]	0.537
Secondary	78 (52.7%)	70 (47.3%)	1.11 [0.372 to 3.34]	0.847
higher	24 (72.7%)	9 (27.3%)	2.67 [0.728 to 9.76]	0.139
Employment				
not working	139 (45.3%)	168 (54.7%)	1.00	
working	74 (54.8%)	61 (45.2%)	1.46 [0.99 to 2.20]	0.065
Marital status				
union/married	181 (48.9%)	189 (51.1%)	1.22 [0.72 to 2.07]	0.456
Single	29 (43.9%)	37 (56.1%)	1.00	
Father education				
none	4 (40%)	6 (60%)	1.00	
primary	65 (44.5%)	81 (55.5%)	1.2 [0.33 to 4.45]	0.781
Secondary	61 (53.5%)	53 (46.5%)	1.73 [0.46 to 6.45]	0.417
higher	24 (68.6%)	11 (31.4%)	3.27 [0.77 to 13.99]	0.110
Residence				
Urban	116 (57.4%)	86 (42.6%)	1.98 [1.36 to 2.91]	** < 0.001**
Rural	95 (40.4%)	140 (59.6%)	1.00	
Household sanitation				
open defecation	76 (37.6%)	126 (62.4%)	1.00	
Sanitation	136 (56.9%)	103 (43.1%)	2.19 [1.49 to 3.21]	** < 0.001**
Parity				
0	64 (47.1%)	72 (52.9%)	1.71 [0.80 to 3.62]	0.161
1–4	136 (50.2%)	135 (49.8%)	1.94 [0.95 to 3.94]	0.068
5 +	13 (34.2%)	25 (65.8%)	1.00	
Pregnancy				
Unplanned	103 (40.9%)	149 (59.1%)	1.00	
Planned	74 (64.9%)	40 (35.1%)	2.68 [1.69 to 4.24]	** < 0.001**
Previous contraceptive use				
Yes	46 (51.1%)	44 (48.9%)	1.16 [0.72 to 1.86]	0.549
No	132 (47.5%)	146 (52.5%)	1.00	

*Abbreviations*: *COR* Crude odds ratio, *CI* Confidence interval

1.00 Reference categories

The results indicate that in the unadjusted analysis the crude odds (COR) of adequate ANC utilization increased approximately twice for women who were within the age group of 20–34 years (COR 2.25; 95% CI: 1.37- 3.70) and 35 years and above (COR 2.12; 95% CI: 1.13–3.98) compared to women in the 14–19 age group. The odds of having adequate ANC service utilization increased approximately twice for women from households with sanitation (COR 2.19; 95% CI: 1.49–3.21) and living in urban areas (COR 1.98; 95% CI: 1.36–2.91) and increased 2.68 times for women who planned the pregnancy (COR 2.68; 95% CI: 1.69 to 4.24).

### Antenatal care utilization – Multivariable analysis

In Table 4, the results obtained from the multivariable logistic regression model are depicted, show the adjusted associations strength between the factors that remained in the final model and ANC utilization. The results obtained from the multivariable logistic regression model, showed the adjusted (AOR) association strength between the factors that remained in the final model and adequate ANC service utilization.

**Table 4:** Multivariable logistic regression analyses of the determinants associated with adequate ANC utilization among pregnant women admitted at Hospital Dr Ayres de Menezes, Sao Tome & Principe

Variable		AOR	95% CI	*p*-value
Age	20–34	2.28	1.28 to 4.04	0.005
	35 +	2.51	1.21 to 5.20	0.013
	14-19^a^			
Residence	Urban	1.98	1.28 to 3.06	0.002
	Rural^a^			
Pregnancy	Planned	2.59	1.60 to 4.18	<0.001
	Unplanned^a^			

*Abbreviations*: *AOR*, adjusted odds ratio; *CI *confidence interval

^a^Reference categories

In the multivariable logistic regression, mothers’ age, residence, and pregnancy type of planning continued to have an association with adequate ANC service utilization. The results indicate that women in the 20–34-year age group (AOR 2.28; 95% CI: 1.28–4.04; *p* = 0.005) and those 35 years old or above (AOR 2.51; 95% CI: 1.21–5.20; *p* = 0.013), living in urban areas (AOR 1.98; 95% CI: 1.28–3.06; *p* = 0.002) and with a planned pregnancy (AOR 2.59; 95% CI: 1.60–4.18; *p* < 0.001) are more likely to have adequate ANC service utilization. A *p* value = 0.810 was obtained by the Hosmer–Lemeshow test indicating that the model was well calibrated.

## Discussion

ANC coverage in STP is a success compared to other SSA countries [10, 16, 19–21]. Attendances are extremely high for a low-resource country, as we found that 85.1% of the participants had four or more visits when rates reported in SSA are approximately 62% [17]. However, this high number of contacts are not equivalent to provision of good practice interventions as this study highlights that more than half of the pregnant women enrolled doesn´t have at least one evidence-based screening test, such as hemoglobin, urine, and obstetric ultrasound. Adequate utilization, meaning that a pregnant woman had four or more ANC contacts, an early booking, had one obstetric ultrasound, and tested for hemoglobin and urine at least once, was only meet by 47.9%.

Our univariable analysis revealed some associations with the factors known to influence ANC utilization with a significant association between women’s age and adequate ANC utilization, as shown in other studies [1, 8, 13]. For example, the higher the age of the expectant mother, the more she would adequately utilize ANC [1, 13]. The same is true for adolescent girls (14–19 years) who are more likely to have partial ANC utilization, probably due to lack of adequate knowledge of essential aspects of ANC service and misconceptions compared to older women [18, 19]. Other reasons for this behavior may be delay in recognizing of the pregnancy status, shame, or negative feelings towards an unwanted pregnancy [12, 18–20]. These findings are similar to those published for other countries in SSA, as it is known that a significant number of pregnant adolescents do not access or do not adequately use ANC services [18] Addressing adolescents´ special pregnancy needs is essential to improve maternal and newborn health in the country [12, 18, 19].

Participants’ educational level was also found to be a strong predictor of adequate ANC utilization. Mothers-to-be who had higher education were more likely to have adequate ANC utilization than their lower educated counterparts. This is not counter-intuitive because previous studies in low-resource countries have reported that the educational level of women increases the odds of having four or more ANC contacts [17, 20–23]. Nonetheless, it should be said that 5.4% of this study´s expectant mothers had less than 16 years old and 14.7% between 17 and 19, meaning that almost 20% of the participants were not old enough to reach a higher education opportunity. The major cause of health inequality in ANC coverage has been strongly associated with a lack of knowledge together with cultural, social, and religious factors [22]. Therefore, reinforcement of women´s education is crucial, as it promotes a better knowledge of maternal and newborn health-related issues [13, 24].

Residence area is another factor with influence [13, 23, 25, 26]. The odds of an adequate ANC utilization increase among urban women, as found in similar studies in low-resource countries [1, 14, 22, 25–27]. In Sao Tome and Príncipe all rural areas have one district maternity available with basic obstetric care and pregnancy follow-up. Thus, different from other studies, this cannot be explained by the lack of these infrastructural facilities or in terms of a far distance. According to published data, in STP, 74% of households have access to health services within 30 min or less, although there are discrepancies between urban areas (87%) and rural areas (59%) [9]. These disparities could be associated with less access to information on pregnancy-related issues compared to their urban counterparts. Household with sanitation showed a significant relationship with ANC utilization, as women from these households have higher odds of utilizing ANC adequately than those practicing open defecation [13]. This finding is probably linked to mother´s wealth and indirect financial autonomy for paying pregnancy exams [28–30].

The results further show that the odds of ANC utilization are higher among working women than non-working women, although without statistical significance [13, 31, 32]. This may be also linked to the ability to pay ANC since working women have their own financial autonomy and are not dependable on their husband/partner or family [29].

Moreover, the rate of adequate utilization of ANC is higher among women with a planned pregnancy. The possible reason might be that pregnant women with a planned pregnancy are much more cautious and eager to know their pregnancy progress than those who had unplanned or unintended pregnancy [21, 29]. Usually, planned pregnancies are safer for the mother and deliver healthier babies, hence, the awareness and the capacity to choose to become pregnant must be a reality to be reinforced [22, 29, 33, 34].

A previous use of contraceptives was found to be associated with adequate ANC utilization, although no statistical significance was established. In STP, most women use traditional (natural) family planning methods as part of a system of traditions that is passed down from generation to generation through the teaching of certain beliefs [9, 10]. We found that only twenty percent of mothers-to-be had a previous contraceptive use, highlighting the need to reinforce the importance of family planning in the country. Incentives should also be made for spacing children as this can reduce mortality among under-fives by 10% and among pregnant mothers by 32% [22]. Thus, family planning services should be enhanced as it promotes small family size, improves child survival, and reduces sibling competition for scarce family and maternal resources [22, 24, 35–37].

These risk factors were also analyzed in other studies conducted by the authors within the context of a broader research on the causes and risk factors contributing to neonatal mortality and adverse birth outcomes in Sao Tome & Principe [38–42].

All the previous findings were obtained in the univariable study. However, in the multivariable analysis only some of the factors remained in the final model. Accordingly, determinants of an adequate ANC utilization in this study were maternal age greater than twenty years (sociodemographic characteristic), urban residence (community level characteristics), and a planned pregnancy (pregnancy related characteristic).

### Strengths and limitations of the study

This study included data with a total number of pregnant women similar to the one recently published for the country in 2019 Multiple Indicator Cluster Survey (MICS) from UNICEF [10]. In contrast to MICS, our study is not the object of recall bias because the data were gathered during the current pregnancy through interviews and data abstraction from ANC pregnancy cards.

Another main strength is that this study included essential routine screenings (obstetric ultrasound, hemoglobin, and urine tests) in addition to the number of contacts per pregnant woman and an early booking during the first trimester. In contrast to most studies in SSA countries that only analyze ANC utilization in terms of the ANC number of contacts and/or timing [12, 13].

This study was conducted in the capital city and may overestimate actual ANC visits as attitudes and practices may differ from rural areas in terms of access to health care, health worker motivation and training, and availability of health services. Factors such as women´s autonomy and the impact of media exposure were not assessed in this study, missing out some determinants associated to ANC utilization, such as sociocultural beliefs and convictions that sometimes dissuade women from accessing the antenatal care services [14].

### Future studies

Further research is needed to determine the effects of traditional, sociocultural, religious, and other related practices at the community-level on the utilization of ANC services. Another important subject would be to determine the extent to which real family planning counseling takes place during ANC visits.

## Conclusion

Less than half of the pregnant women reached adequate ANC utilization, highlighting the challenge still ahead for ANC optimization in Sao Tome & Principe. The present study showed that the determinants of an adequate ANC utilization were maternal age greater than twenty years, urban residence, and a planned pregnancy. Understanding pregnant women´s determinants of ANC utilization in view of improving it is an excellent bridge to achieve its optimization. Measures such as raising awareness of the importance of ANC screenings completion, engaging the more vulnerable women in earlier utilization of family planning services, and promoting each women empowerment for a pregnancy plan are key strategies to improve neonatal health outcomes in the country.

## Data Availability

The datasets used and/or analyzed during the current study are all available within the manuscript itself.

## Abbreviations

STP: Sao Tome & Principe
HAM: Hospital Dr. Ayres de Menezes
OR: Odds Ratio
CI: Confidence Interval
SDG: Sustainable Development Goal
ANC: Antenatal care
MICS: Multiple Indicator Cluster Survey
WHO: World Health Organization
FANC: Focused Antenatal Care
